# New extensive sampling transforms our understanding of global marine diversity

**DOI:** 10.1371/journal.pbio.3003440

**Published:** 2025-10-31

**Authors:** Jonathan Belmaker

**Affiliations:** 1 School of Zoology, George S. Wise Faculty of Life Sciences, Tel Aviv University, Tel Aviv, Israel; 2 Stenhardt Museum of Natural History, Tel Aviv University, Tel Aviv, Israel

## Abstract

Scientists still have a very limited understanding of marine biodiversity. This Primer explores a new study in PLOS Biology that uses unprecedented global environmental DNA (eDNA) sampling to reveal the extent of our ignorance.

## Teaser

Scientists still have a very limited understanding of marine biodiversity. A new study in *PLOS Biology* using unprecedented global environmental DNA (eDNA) sampling reveals the extent of our ignorance and charts the way forward.

The marine environment is vast. While scientific endeavors to decipher its secrets are increasing, e.g., through multi-national collaborations for data integration and mobilizations [1,2], we are still, literally, just scratching the surface. A recent study in *PLOS Biology* by Sanchez and colleagues summarized an enormous sampling campaign spanning 6 years and nearly one thousand samples across the most remote sections of the world’s oceans [3]. The new data vastly extends our understanding of the geographic and environmental boundaries of most marine fishes examined. The new understanding of how marine species are distributed is critical for predicting how marine ecosystems will respond to rapidly changing climates, habitat modifications, and fishing exploitation.

Most global studies that characterize species distributions are based on large compilations of observations (e.g., GBIF and OBIS), derived from varied resources such as scientific expeditions, national biodiversity data centers, and citizen science initiatives. While such sources are known to be biased, underrepresenting certain regions and specific habitats (e.g., the pelagic realm and deeper ocean layers [4]), in practice, there have been few alternatives. Recently, environmental DNA (eDNA) has emerged as a novel way to estimate species occurrences accurately and with minimal disturbance. eDNA is an exciting method that uses genetic material released by organisms into the seawater to identify their presence. By filtering and extracting DNA fragments from seawater samples, researchers can identify the presence of marine species without physically capturing them. However, it remains unclear to what extent this method has the potential to transform our knowledge on ocean diversity.

**Fig 1:**
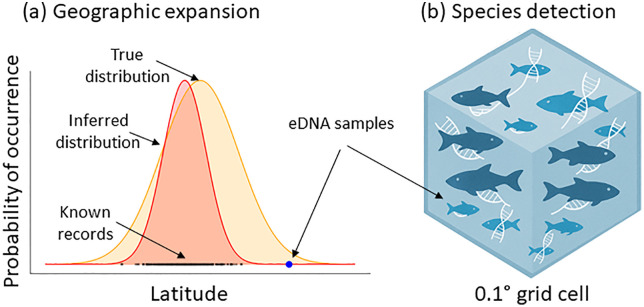
Schematic illustration of how extensive sampling allows better estimates of species geographic distributions and the detection of new species. **(a)** The currently inferred distribution of species based on known records (red, records illustrated using black dots) is substantially expanded to better approximate the true distribution (yellow) when using new eDNA sampling at a global extent. **(b)** These eDNA samples can also detect new species records (light blue fishes) within ocean grid cells, especially species that are small and rare. Illustration prepared with the assistance of GPT5.0.

Sanchez and colleagues [3] have undertaken the most spatially extensive eDNA sampling effort to date. During 6 years of expeditions, they collected nearly one thousand eDNA samples from the most extreme corners of the globe—from remote tropical islands to polar oceans. This new data allowed them to compare what was previously known about global fish distributions with what can be gained by this emerging method (Fig 1). The authors show how eDNA can be used to identify new species records even in locations that have been extensively studied using other methods. These new records belong to species that are typically rare and small (cryptobenthic) and thus not easily detected by conventional sampling. Moreover, the authors have also identified many new records from completely new locations. In fact, new records that substantially increase our estimates of species ranges (new occurrences increasing area of occupancy by at least 10 km) were found for over 93% of the species. These new records were found across the board—for species common and rare; small; and large.

These new records have important implications for understanding changes to marine life. For example, estimates of how far and how fast species ranges are shifting due to climate change [5], or how fast ranges are declining due to fishing [6], rely on accurate estimates of where species occur. A new record may thus either represent a genuine change, e.g., a recent range shift, or simply a location previously gone unnoticed. Without sufficiently accurate estimates of where species occur, we cannot tease these apart. The results by Sanchez and colleagues [3] are a sobering realization of how little we know about marine biodiversity, even for well-known groups with economic importance such as fishes.

The sheer scale of the data compilation effort and the corresponding amount of new records make this study a strong case for the need for more, and better, sampling. However, Sanchez and colleagues [3] also use this information to predict the number of new species occurrences that can be gained with additional eDNA samples, and identify areas where additional sampling is likely to provide the most added benefit. The authors find that a fairly light sampling effort of 10 eDNA samples per grid cell of 0.1° would detect 24 additional fish species on an average, but up to 98 species in previously unsampled tropical areas. This information provides a clear opportunity, and challenge, to the international community to promote a collaborative and coordinated global sampling campaign.

While the utility of eDNA sampling is clearly demonstrated by this study, a few important limitations must be acknowledged. First, reaching the most remote ocean locations is challenging—regardless of the sampling method. Hence, repeating and extending such a sampling expedition is unlikely to be an easy task. Second, eDNA relies on good reference DNA libraries. While this information may exist for many fishes, other marine taxa are far from having sufficient DNA library coverage in most regions. Finally, eDNA may not reflect true local abundances due to many issues related to rates of mixing and transport of DNA fragments in the ocean currents, varying eDNA degradation rates, and the unknown relationships between true fish density and eDNA concentrations. This means eDNA sampling is not a silver bullet, and that employing multiple sampling methods will be required to track marine biodiversity change, including surveys based on citizen science [7]. Together, we may be able to finally understand where marine diversity is actually located and, consequently, to plan better ways to conserve it.

## Abbreviation

**:**
eDNA: environmental DNA
